# A Primer on Data Analytics in Functional Genomics: How to Move from Data to Insight?

**DOI:** 10.1016/j.tibs.2018.10.010

**Published:** 2019-01

**Authors:** Piotr Grabowski, Juri Rappsilber

**Affiliations:** 1 Bioanalytics, Institute of Biotechnology, Technische Universität Berlin, 13355 Berlin, Germany; 2 Wellcome Centre for Cell Biology, University of Edinburgh, Edinburgh EH9 3BF, UK

**Keywords:** data integration, data science, functional genomics, machine learning, systems biology

## Abstract

High-throughput methodologies and machine learning have been central in developing systems-level perspectives in molecular biology. Unfortunately, performing such integrative analyses has traditionally been reserved for bioinformaticians. This is now changing with the appearance of resources to help bench-side biologists become skilled at computational data analysis and handling large omics data sets. Here, we show an entry route into the field of omics data analytics. We provide information about easily accessible data sources and suggest some first steps for aspiring computational data analysts. Moreover, we highlight how machine learning is transforming the field and how it can help make sense of biological data. Finally, we suggest good starting points for self-learning and hope to convince readers that computational data analysis and programming are not intimidating.

## Highlights

High-throughput technologies are now widely used in the life sciences field and are producing ever-increasing amounts and diversity of data.

While many laboratories and even undergraduate students generate high-throughput data, analyzing these results requires a skill set that is traditionally reserved for bioinformaticians.

Learning to program using languages such as R and Python and making sense of the vast amounts of available omics data have become easier, thanks to the multitude of available resources. This can empower bench-side researchers to perform more complex computational analyses.

Tools such as KNIME or Galaxy (together with a growing number of tutorials and courses) have been crucial in providing simple user interfaces to conduct complex analyses under the hood, making the ‘big data’ revolution accessible to biologists.

## Can a ‘Traditional’ Biologist Handle Big Data?

Biologists are facing an exciting yet challenging time with the increasing availability of high-throughput data sets that need to be analyzed and understood. These omics data sets can be either integrated with self-generated data or reanalyzed independently. In the former case, the extra dimension provided by the new data can help generate additional hypotheses on biological systems or support hypothesis validation. In the second case, one can consider published data from a different perspective than that intended in the original study, integrating additional data sources, to make new discoveries without having to invest the time and funds in acquiring new data. Reanalysis and repurposing of published data is a growing trend [1]. The field of biological sciences is expecting a rise in specialists in data integration and interpretation.

Integrative multiomics is a rapidly growing field, as reviewed by 2, 3. In addition, one of the exciting fields with increasing amounts of impact and deposited data are the single-cell technologies which encompass genomics, transcriptomics, and epigenomics 4, 5. These technologies can be especially powerful when combined with other types of data [6].

The term ‘multiomics’ refers to the process of integrating data from different high-throughput technologies. Examples of such combinations are as follows:

• Genomics + transcriptomics, often used in expression quantitative trait loci (eQTLs) studies, which can elucidate genomic variants that are important for cellular functions and disease.
• Transcriptomics + proteomics, relating how the transcriptome is shaping the proteome to the possible post-transcriptional and post-translational mechanisms governing this process, as reviewed in [7].
• Proteomics + metabolomics, correlating differences in protein levels with the metabolites they regulate, synthesize, or degrade 8, 9.
• Epigenetics + transcriptomics + proteomics, particularly how the regulatory state of the genome influences gene expression [10] or to obtain a holistic view of stem cell differentiation [11].
• Phenomics + genomics + transcriptomics, relating external phenotypic traits to genetic sequences and gene expression, which can be helpful in plant biotechnology, for example, [12].

Analyzing and making sense of such large data sets can be challenging. A natural ally for this task is machine learning, which is becoming the go-to method for developing analytical workflows for multivariate omics data. It can be used to build models for data classification (e.g., to separate healthy and sick patients or protein members of different subcellular components), to cluster data into separate groups, reduce the dimensionality of the data set for visualization, and perform missing value estimation. However, using machine learning requires more knowledge and experience than performing basic statistical hypothesis testing in Excel-like spreadsheet environments. One has to understand the basic concepts to avoid producing nonsensical results.

Moreover, data processing, integration, and modeling require some degree of programming skills. For this reason, analyzing such data and using machine learning have traditionally been delegated to computer-savvy experts. This often prohibits any hands-on contact from the domain specialists with their data, especially in heavily wet laboratory-oriented fields. Programming languages such as R and Python offer unlimited power for analysis but require some level of fluency in writing instructions and knowing relevant functions and packages. Knowing at least one analytics platform is paramount to performing any integrative omics study.

**Figure 1:**
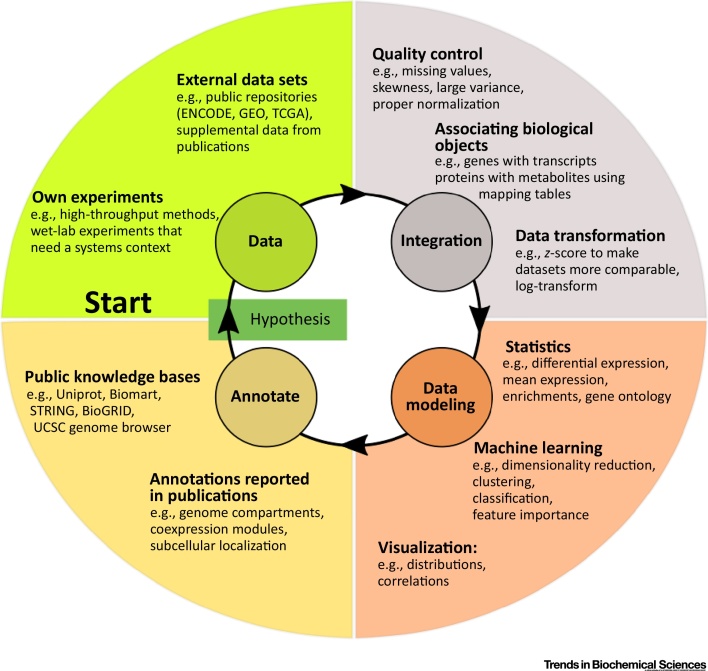
Basic High-Level Flow of Omics Data Analytics in the Life Sciences Field.

This opinion paper is a conceptual primer aimed mainly at graduate students, PhD students, and postdoctoral researchers who want to start their journey into computational data analysis, but are not sure about the overall breadth of the field, which are the important first steps to take, and what resources are available. We propose a meta-level workflow consisting of four elements: (i) obtaining processed data from public repositories, which can be used alone or in conjunction with self-generated data, (ii) hands-on manipulation and processing methods for large data sets, (iii) using statistics and machine learning to find significant differences and/or relationships, (iv) accessing knowledge and annotation databases to help extract novel insights (Figure 1). Finally, we give some tips on learning resources that might be helpful to start one’s journey into integrative data analytics and machine learning.

## Where to Find Publicly Available Data?

The volume of biological and biomedical data deposited into public repositories and databases is vast and growing every week. This offers a valuable resource to those who are able to navigate them. These data are free and instantly available. This can allow for rapid testing of one’s ideas without delays associated with experiment planning and data acquisition. Some of these repositories are listed in Table 1.

**Table 1:** Summary of Large Data Repositories for Omics Analytics

Repository	Data type	Link
Gene Expression Omnibus	Gene expression, noncoding RNA profiling, epigenetics, genome variation profiling	www.ncbi.nlm.nih.gov/geo/
ENCODE	Epigenetics, gene expression, computational predictions	www.encodeproject.org
ArrayExpress	DNA sequencing, gene and protein expression, epigenetics	www.ebi.ac.uk/arrayexpress/
European Genome-Phenome Archive^a^	Various omics with phenotype data (biomedical studies)	https://ega-archive.org
PRoteomics IDEntifications (PRIDE), ProteomeXchange	Proteomics, protein expression, post-translational modifications	www.ebi.ac.uk/pride/archive/ http://www.proteomexchange.org/
1000 Genomes	Genome sequences, sequence variants	www.internationalgenome.org
MetaboLights	Metabolomics	www.ebi.ac.uk/metabolights/
GTEx^a^	Gene expression (microarrays and RNA-seq), genome sequences	www.gtexportal.org
National Institutes of Health/National Cancer Institute (NIH/NCI) Genomic Data Commons	Gene expression, epigenetics, miRNA-seq (focus on cancer)	https://portal.gdc.cancer.gov
NIH dbGaP^a^	Genotypes, gene expression, epigenetics, phenotypes	https://www.ncbi.nlm.nih.gov/gap
cBioPortal	Focused on cancer, contains data on gene copy numbers, gene and protein expression, DNA methylation, and clinical data	http://www.cbioportal.org
Single Cell Expression Atlas	Single-cell gene expression (RNA-seq)	https://www.ebi.ac.uk/gxa/sc/
RIKEN SCPortalen	Single-cell gene expression (RNA-seq)	http://single-cell.clst.riken.jp/

a Needs granted access for individual-level data.

The National Center for Biotechnology Information’s (NCBI) Gene Expression Omnibus (GEO) is an example of such a repository which, as of May 2018, contains nearly 4500 curated data sets on gene expression, epigenetics, and genome variation profiling. A useful Web resource for GEO-deposited data is the ARCHS^4^ (https://amp.pharm.mssm.edu/archs4/) from the Ma’ayan laboratory [13], which provides access to processed gene expression tables from the raw data deposited in GEO and Sequence Read Archive (SRA). Of note, the main difference between GEO and SRA is that GEO contains processed data while the raw data (such as FASTQ files from a sequencing run) are deposited into the SRA. This means that if one is looking for ‘ready-to-use’ gene expression tables, one should search the GEO.

The Encyclopedia of DNA Elements (ENCODE, [14]) consortium provides a high-quality multiomics data resource for human, mouse, worm, and fruit fly models. It contains data on gene expression, epigenetics, and 3D genome conformations that are generated through a variety of technologies. In addition, the ENCODE consortium provides computational annotation such as predicted DNA regulatory elements.

ProteomeXchange [15] stores published proteomics data sets from over 9000 projects, covering a multitude of species. The data sets tagged ‘biological/biomedical’ pertain to the general research audience, or can be tagged ‘technical’, if they are more relevant to the specialized proteomics community. Sometimes, the deposited data are in the so-called raw format only, which would require a preliminary processing step using proteomics software before it can be interpreted. However, one can typically find the processed protein or peptide quantification tables in the accompanying manuscript.

The European Genome-Phenome Archive (https://ega-archive.org) [16] offers a large collection of biomedical omics data from multiple studies. However, as is often the case with medical databases containing sensitive patient information, one has to apply to gain access via official channels.

The Genotype-Tissue Expression (GTEx) Consortium Portal [17] (www.gtexportal.org) stores omics data from a panel of 53 human tissues from densely genotyped donors. The combination of gene expression data with genomic variants and patient information greatly facilitates eQTL studies.

The database of Genotypes and Phenotypes (dbGaP) [18] (https://www.ncbi.nlm.nih.gov/gap) is a database archiving data about interactions of human genotype and phenotype. The data types encompass DNA variation, single-nucleotide polymorphism assays, DNA methylation, copy number variation, and gene expression profiling using technologies such as RNA-seq and microarrays. Those are linked to phenotype data such as disease-related clinical status.

Single Cell Expression Atlas (https://www.ebi.ac.uk/gxa/sc/) and SCPortalen (http://single-cell.clst.riken.jp/) are repositories for data acquired using single-cell technologies, such as single-cell RNA-seq.

Aside from technology- and domain-specific resources, initiatives now exist for the global integration of omics data sets according to the FAIR principles (‘findable, accessible, interoperable, and reusable’). The biggest such initiative is the Omics Discovery Index [19] (https://www.omicsdi.org/), which provides an open-source platform for discovery, access, and dissemination of published omics data, and currently integrates 11 repositories. An interesting feature available on this platform is the ‘similar dataset’ section, which can be used to search for other data sets that are conceptually related, similar to recommended products in online stores.

## How to Analyze Big Data Sets?

After downloading the data set, the next step is to carry out an integrative analysis. Initially, this process involves a series of data quality checks (such as looking at data distributions and ranges or looking for any missing values) and joining of data sets based on common ID systems (usually requires downloading ID translation tables). Subsequently, one can then perform the desired statistical analyses or run machine learning workflows and/or annotate the data using external knowledge bases. All of these steps require appropriate software.

Next-generation sequencing data often need processing before they can be represented in, for example, expression table. To help with these steps, the Galaxy platform [20] offers powerful solutions. It was developed with user-friendliness and simplicity in mind to allow nonspecialists to handle genome and transcriptome data using a simple Web-based user interface. Importantly, the user does not have to worry about providing enough computational resources as these are provided by many Galaxy-hosting institutions. Alternatively, a Galaxy server can be quickly set up on a local server.

KNIME [21] is an accessible entry point for time-constrained biologists or for those daunted by programming. It is a graphical user interface (GUI) analytics environment that offers a ‘point and click’ alternative to classical programming. One can create node-based workflows in which each node is a function that takes in a certain object (e.g., gene and protein expression tables), processes it, and outputs the results (e.g., combined expression data as one matrix). This modular approach offers flexibility and allows one to be creative while keeping the entire workflow easy to follow and reproducible. The ‘Node Guide’ section of the KNIME Web page is a great starting point with many examples and downloadable workflows (https://www.knime.com/nodeguide). Moreover, a hub for bioinformatics problems was recently developed to share KNIME workflows for biological data processing and analysis (https://cibi.uni-konstanz.de/hub). More information on using KNIME in the life sciences field can be found here [22].

Choosing between GUI-based analytical platforms such as KNIME or ‘classical’ programming languages is a personal matter. KNIME offers a lot of ready-to-use functionalities to combine using a GUI. While this allows for a quicker start, it also has limitations (e.g., the user is limited only to the implemented nodes). Programming languages such as R and Python offer much more flexibility for data analytics and are considered the standard tools of trade in research and industry. The choice between R and Python is mostly related to personal preferences. However, it might be more productive to start with a language that is more commonly used in one’s professional environment as this enables code sharing and hands-on help from colleagues. Both R and Python offer very versatile and powerful analytical environments. Until recently, R was a more popular choice among biologists as it had more mature libraries for biological data (including the popular Bioconductor package repository). This is changing now, as the statistical and biological analytics suite for Python is being constantly expanded. Both languages have a syntax that is relatively easy to learn and there are no major speed differences between the two when it comes to typical data operations. One advice is to simply try both for a short period and see which language is a better fit.

An important aspect of productive analytical programming is selecting the integrated development environment (IDE). IDEs are programs that help programmers to write code by providing access to coding tools, an interactive programming console, plotting areas and variable inspectors. Analyzing data using R and Python without an IDE is more challenging and we highly recommend using one such as RStudio for R and PyCharm or Spyder for Python.

One has to be cautious when integrating data from many sources such as multiple technologies and even laboratories. Most quantification technologies require proper data normalization procedures, for example, using a control sample that can take into account measurement noise related to a given platform. It is advisable to work using normalized values or to calculate them, if both the sample of interest and a control are available in the repository. In the worst case, the observed signal in the data might be simply technical noise and not genuine biological change, due to lack of proper normalization. Furthermore, it is important to understand how a given unit is being used in the field. For example, RNA-seq expression values fragments per kilobase of transcript per million mapped reads (FPKM) and reads per kilobase of transcript per million mapped reads (RPKM) are typically used for visualization and ranking. However, one should avoid using those widely used units for differential gene expression analysis [23]. Good practices for other types of data, such as ChIP-seq, can be found elsewhere [24]. We strongly recommend familiarizing oneself with the way analyses are carried out in respective fields prior to downloading and integrating omics data sets.

## How Can Machine Learning Help You with Your Data?

Dealing with big data sets is not easy. To address this, one of the tools that has become very popular in the life sciences field is machine learning. In brief, machine learning is a collective term for computer algorithms that iteratively fit a predictive model to the observed data. This model can then be generally applied to predict properties of yet unencountered data, as long as they can be described by the same features. The breadth and depth of this dynamic field have been extensively reviewed 25, 26, 27. Here, we will focus on the practical basics regarding the usefulness of machine learning in biology and provide an example of a machine learning workflow design in Box 1.

Box 1 **Machine Learning in Biology. How to Approach a Machine Learning Analysis for Biological Questions?**

Planning a machine learning analysis can be an overwhelming task for a researcher lacking computational experience. In Figure I, we divided an example classification workflow (mitochondrial protein prediction) into separate stages while emphasizing important questions that one should consider at each stage.

First, one has to define the target variable of interest and think about what can represent the positive and negative examples of the target.

Second, one has to carefully assemble a training set (for supervised methods). Selecting only confident positive and negative examples is essential for the quality of the final analysis. One can perform a manual literature search or take examples (such as proteins) on which there is largest agreement between the different databases.

Third, the input data used by the algorithm should contain enough positive and negative training examples. Importantly, machine learning should not be treated as remedy for low-quality data. The classical statistical rule (crap-in, crap-out) applies to machine learning as well.

Subsequently, one should select a proper algorithm for the task. This step depends on the target type (classification vs. regression), number of available training data, and technicalities such as presence of missing values.

Finally, the resulting class probabilities (or predicted continuous target values in regression) should be manually evaluated. At this stage one can check if there are any overfitting or underfitting problems and evaluate the workflow’s performance using statistics such as mean accuracy (for classification) or mean-squared error (for regression) using left-out (test) data. Such statistically evaluated ranking can then be used with external annotation databases such as STRING or Enrichr and further validated in the wet laboratory or used to build new hypothesis for more computational exploration.

Alt-text: Box 1

**Figure I:**
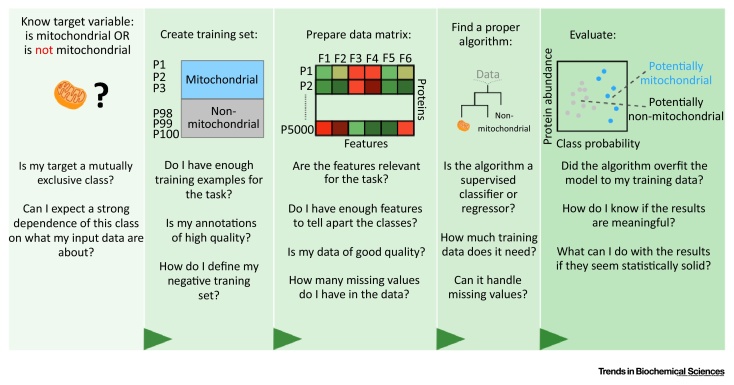
Planning a Machine Learning-Based Analysis Requires Careful Consideration at Each Stage of the Analysis. We listed the most general elements of designing such workflow using mitochondrial protein classification task as an example. However, same thinking patterns apply to regression tasks or for feature importance analysis.

In general, machine learning approaches are divided into two main classes: supervised and unsupervised algorithms. Supervised learning algorithms build a mathematical description (a model) of how a combination of features, such as a gene expression values, relates to some target variable, such as ‘is important in cancer progression’. These models can then be used to predict the target variable (classes) for data that the model has not yet encountered. An example of this is predicting subcellular localization of proteins 28, 29, 30. Here, one has to first feed the algorithm a data set together with high-quality annotation, such as proteins assigned to known subcellular compartments (a training set), on which to train the model. After this process, the trained classifier can be used to assign subcellular localizations of other proteins in the data set. Similar to the classification task, a supervised machine learning algorithm can be trained to predict continuous values instead of classes (i.e., perform regression), such as chromatographic retention times of peptides [31] or predicting gene expression levels using data on epigenetics and genomic features [32].

Unsupervised approaches, as opposed to supervised approaches, do not require a prespecified target variable of interest. Instead, this broad group of algorithms can help find (and exploit) structure in the data. An example of such approach widely used in biology is data clustering, which allows to group observations according to their properties. One can imagine a panel of samples that are not clearly distinguished by some binary classification (like cancer/healthy), but rather having various genomic mutations. Having obtained protein expression profiles for each of the samples, one can use an unsupervised approach to see which of those mutations behave similarly to one another. A typical algorithm used in such situation is hierarchical clustering which generates a dendrogram in the process. Cutting this dendrogram at a selected height results in formation of distinct clusters. These clusters can be then analyzed for functional enrichments (described in more detail in the next section). Unsupervised approaches have been useful for finding groups of coregulated proteins in cancer [33], finding cobehaving mRNA and miRNA modules in time-series data [34], or finding coexpressed genes in many samples 10, 35.

Yet another type of unsupervised algorithms allows for dealing with high-dimensional data, for example, when one is interested in visualizing these or detecting outliers, by performing dimensionality reduction. One of the most popular algorithms for this task is principal component analysis. A good description of how it works and its applications in biology can be found elsewhere [36].

Another interesting application of machine learning is identification of novel predictive features for an observed phenotype (known collectively as ‘feature importance analysis’ or ‘feature selection’). Here, machine learning is first used for a classification or regression task as described earlier. However, during this process, many algorithms can inform the user about which of the used features were the most important for a given task. Subsequently, one can look at how well the selected features correlate with the target variable. An example of such approach is expanding the model of nonsense-mediated mRNA decay (NMD) [37]. Here, Lindeboom *et al.* looked at levels of NMD in human cancers and developed additional descriptors based on genomic features (such as length of an exon harboring mutation). Using random forest-based regression they could identify which of those new features are important for predicting NMD efficiency, thereby expanding the current model. A short review of such approaches in biology can be found in [38].

Machine learning pipelines can be built using R, Python, and KNIME (among many other languages and platforms). While KNIME offers a great selection of machine learning nodes, including WEKA [39] and H2O (http://docs.h2o.ai/) implementations, it offers less flexibility for pipeline development compared with programming languages such as R and Python. We found that starting with machine learning in KNIME and switching to ‘classical’ programming languages worked best for many of our students. This allowed them to first learn the absolute basics of analytics and subsequently give them more creative freedom.

One of the best places to start using machine learning in R is the ‘caret’ package, which offers functions for data processing, classification and regression algorithms, feature selection, and model evaluation tools. Similar to R, Python offers a powerful machine learning environment: ‘scitkit-learn’ [40]. Moreover, a good place to start one’s journey with machine learning is downloading the Iris data set and following one of the many tutorials for a respective machine learning environment (e.g., http://scikit-learn.org/stable/auto_examples/datasets/plot_iris_dataset.html).

## How to Annotate Results and Generate Hypotheses?

Biological data that have accumulated over the past decades is collated in databases using systems of annotations and ontologies. One can use these external databases (Table 2) to help explain functional relationships between genes or proteins of interest in new data sets. For example, using information about pathways can indicate if observed expression changes are modulating particular cellular functions.

**Table 2:** Summary of Annotation Databases and Postanalysis Tools Helpful in Making Sense of Results in Computational Analytics

Annotation database/Tool name	Description	Link
UniProt	Comprehensive proteomics knowledge base (functions, pathways, sequences, modifications, literature references, ID conversion).	https://www.uniprot.org
BioMart	Gene-centric database with ID conversion, genomic features (such as exons, introns, untranslated regions), sequences, positions of genes in the genome.	https://www.ensembl.org/biomart/martview/
NCBI Genome Data Viewer	A Web tool for exploration and analysis of eukaryotic genome assemblies.	https://www.ncbi.nlm.nih.gov/genome/gdv/
UCSC Genome Browser	A collection of tools for analysis of genomes with a plethora of available data ‘tracks’ such as epigenetic signals and genomic features.	https://genome.ucsc.edu/
StringDB	A database of known and predicted protein–protein interactions. Integrates functional relationship data from various sources.	https://www.string-db.org/
BioGRID	Curated database of physical and genetic interactions based on various experimental sources.	https://thebiogrid.org/
DAVID	Gene Ontology and pathway analysis Web tool for calculation of functional enrichments in lists of genes or proteins.	https://david.ncifcrf.gov/
Enrichr	Web tool for calculating various functional enrichments in lists of genes or proteins.	https://amp.pharm.mssm.edu/Enrichr/
g:Profiler	Web tools for functional profiling of groups of genes or proteins. Contains useful ID conversion and orthology mapping tools.	https://biit.cs.ut.ee/gprofiler/

A popular knowledge base is UniProt (www.uniprot.org), which is a protein-centric resource, annotating the proteomes of many studied species. Swiss-Prot is the manually curated part of the database, offering high-quality annotation. It should be preferred over the electronically generated TrEMBL annotation for functional genomics analyses. The ‘Retrieve/ID mapping’ tool by UniProt allows mapping of both protein/gene identifiers between different systems (such as RefSeq to UniProt Accession numbers) and query lists of proteins to annotate them with biological properties such as protein sequences, domains, subcellular localization.

BioMart is another widely used database (with a helpful R package biomaRt [41] and a Python library [42]), found at https://www.ensembl.org/biomart/martview/. BioMart offers biological annotation such as genomic coordinates, transcripts and proteins associated with a given gene, sequences, GC content, genetic variants, or protein domains. The genes of interest (the query) are configured in the ‘Filters’ section of the database while the relevant biological information that one may wish to download is configured in the ‘Attributes’ section. The resulting annotated data table can be then saved to disk as a .csv file and integrated into the analytical workflow by matching the gene, transcript, or protein IDs.

For genome-level annotation, NCBI offers the Genome Data Viewer (https://www.ncbi.nlm.nih.gov/genome/gdv/), a tool for exploring eukaryotic genomes. This tool can be used to find positions of genes and annotate the genome track with various types of external data. Another similar tool for genome-level analysis is the UCSC Genome Browser (https://genome.ucsc.edu/), which focuses predominantly on human and mouse genomes and offers vast amounts of functional data integrated in ‘tracks’ that are aligned to a given genome. UCSC Genome Browser can seem overwhelming at first, but the steep learning curve for this tool is worth enduring.

Some online resources offer even more ‘distilled’ levels of biological information. Search Tool for the Retrieval of Interacting Genes/Proteins (STRING) [43] offers a database (https://www.string-db.org) on functional connectivity between genes/proteins. Users can search for interaction networks between genes/proteins of interest or download the entire database. STRING collates an array of biological sources such as biochemical experiments, text mining, and co-expression studies and produces an integrated score. It offers a very simple and fast way to check if a group of genes/proteins is functionally related. Apart from the integrated score, STRING also performs simple Gene Ontology (GO) and Kyoto Encyclopedia of Genes and Genomes (KEGG) enrichment analysis, further aiding hypotheses development. An alternative resource to STRING is the Biological General Repository for Interaction Datasets (BioGRID) [44], which hosts a variety of interaction data for multiple species. Other easy-to-use tools for functional enrichment and pathway analysis are the Gene Ontology-centered DAVID [45], which can help discover biologically important modules after performing differential expression analysis or data clustering. Enrichr [46], available at http://amp.pharm.mssm.edu/Enrichr/enrich, is another tool that takes a list of genes and calculates enrichments in many functional categories such as pathways, ontologies, or transcription-factor binding. Finally, gene set enrichment analysis [47] can help analyze whether an *a priori*-defined group of genes is significantly affected in given biological states.

In addition to knowledge bases that contain annotation for multiple species, there are specialized resources curated by communities that are focused on specific organisms or groups of organisms. Examples include the Saccharomyces Genome Database (https://www.yeastgenome.org/), WormBase aimed at nematodes (https://wormbase.org), FlyBase aimed at *Drosophila* (http://flybase.org), or SubtiWiki focused on *Bacillus subtilis* biology (http://subtiwiki.uni-goettingen.de/).

## Where to Find Help?

Learning to handle large-scale data analysis has become increasingly accessible thanks to numerous resources available on the Internet. Acquiring these specialized skills is no longer limited to hands-on training organized at institutes, but can be done from the comfort of one’s office or home and with exceptional time flexibility. One of the largest providers of such resources are Coursera and edX.org. These commercial platforms offer dozens of courses on programming, statistics, machine learning, and even genomics.

One of the most popular courses on machine learning is the course offered by Andrew Ng (simply called ‘Machine Learning’, found at https://www.coursera.org/learn/machine-learning). This course is a good place to start one’s adventure with machine learning, as the concepts are explained in a very intuitive and math-light way. Another important skill (especially for people interested in using Python and R) is understanding the basic concepts of programming and computer science. One of these courses is the ‘Introduction to Computer Science and Programming Using Python’ offered by the Massachusetts Institute of Technology (MIT) on the edX.org platform. Even though the course is Python based, the concepts learned are transferable to other programming languages, such as R. An advantage of this course is that it is free. In addition to this, a good read is ‘Ten simple rules for biologists learning to program’ [48].

Another worthwhile resource is Coursera’s ‘Statistics with R Specialization’, which is a bundle of courses that teaches statistics and R simultaneously and can be of benefit to anyone who is interested in functional genomic analyses (which are inherently statistics heavy). Aside from commercial providers, there are high-quality online courses from the European Molecular Biology Laboratory/European Bioinformatics Institute (EMBL-EBI), which can be found at www.ebi.ac.uk/training/online/. Here, the spectrum of skills is more concentrated on applied biological problems and specific platforms, such as analyzing RNA-seq data. Moreover, the genomics and biostatistics courses from Rafael Irizarry (found at https://rafalab.github.io/pages/teaching.html) are another high-quality and free learning resource on biological data analytics. For a more general selection of courses on R, Python, and Data Science, one can refer to DataCamp (https://www.datacamp.com/). It offers high-quality courses with a free (albeit limited) membership plan.

Furthermore, specialists in the omics field can be accessed through various forums with specific questions. We strongly encourage referring to those forums when analyzing data. This improves one’s understanding of the data peculiarities and various analytical approaches needed to extract knowledge from the data sets and allows developing all the required skills much faster, while avoiding potential beginner’s mistakes. Biostars (https://www.biostars.org/) and SEQanswers (http://seqanswers.com/) are forums with very active bioinformatics communities and good places to seek help.

## Concluding Remarks

The curricula of most bioscience programs already contain elements of computational data analytics. However, there is a need for increased focus on this subject, to encourage students to complete their degrees with a working knowledge of at least one programming language and statistics. Luckily for those who have already finished their formal education, many learning resources are available that are well-structured and contain high-quality material, while forums offer expert advice to overcome any challenges. The only prerequisite is that one has to be prepared to battle through the initial confusion and understand that the time investment will pay off in the near future. Just do it! (see Outstanding Questions).

Outstanding Questions How can we further improve omics data analytics for wet-laboratory researchers?

How can we enhance data accessibility and annotation so that it can be efficiently reused?

What changes in teaching curricula should be introduced to increase computational competence of students and young researchers?
